# Correction: Intrinsic, pro-apoptotic effects of IGFBP-3 on breast cancer cells are reversible: involvement of PKA, Rho, and ceramide

**DOI:** 10.3389/fendo.2026.1810747

**Published:** 2026-03-25

**Authors:** Claire M. Perks, Carla Burrows, Jeff M. P. Holly

**Affiliations:** IGFs and Metabolic Endocrinology Group, School of Clinical Sciences, Learning and Research Building, Southmead Hospital, University of Bristol, Bristol, United Kingdom

**Keywords:** cell survival, apoptosis, IGF-independent role, insulin-like growth factor, nuclear binding partners of IGFBP-3, IGFBP-3 interacting proteins, cell penetration peptides, insulin-like growth factor binding protein-3

There was a mistake in **Figure 3** as published. The different treatments, illustrated in **Figures 3B, C**, were performed in one experiment, and the subsequent cell lysates were then run on the same gel. To ensure the most efficient use of costly reagents, all lanes on the gels were used with samples from different treatments of the cells, but not all the results from the different treatments were relevant for this manuscript and so we acknowledge that these were cropped out to produce just the relevant data shown in **Figures 3B, C**. We also wish to acknowledge that we used inconsistent terminology for IGFBP-3 in **Figures 3B, C**, BP3 in **Figure 3B** and IGFBP-3 in **Figure 3C**. These are now consistently written as IGFBP-3. Also, we used SFM and CT interchangeably and now it is consistently written as CT. The corrected **Figure 3** appears below.

**Figure 3:**
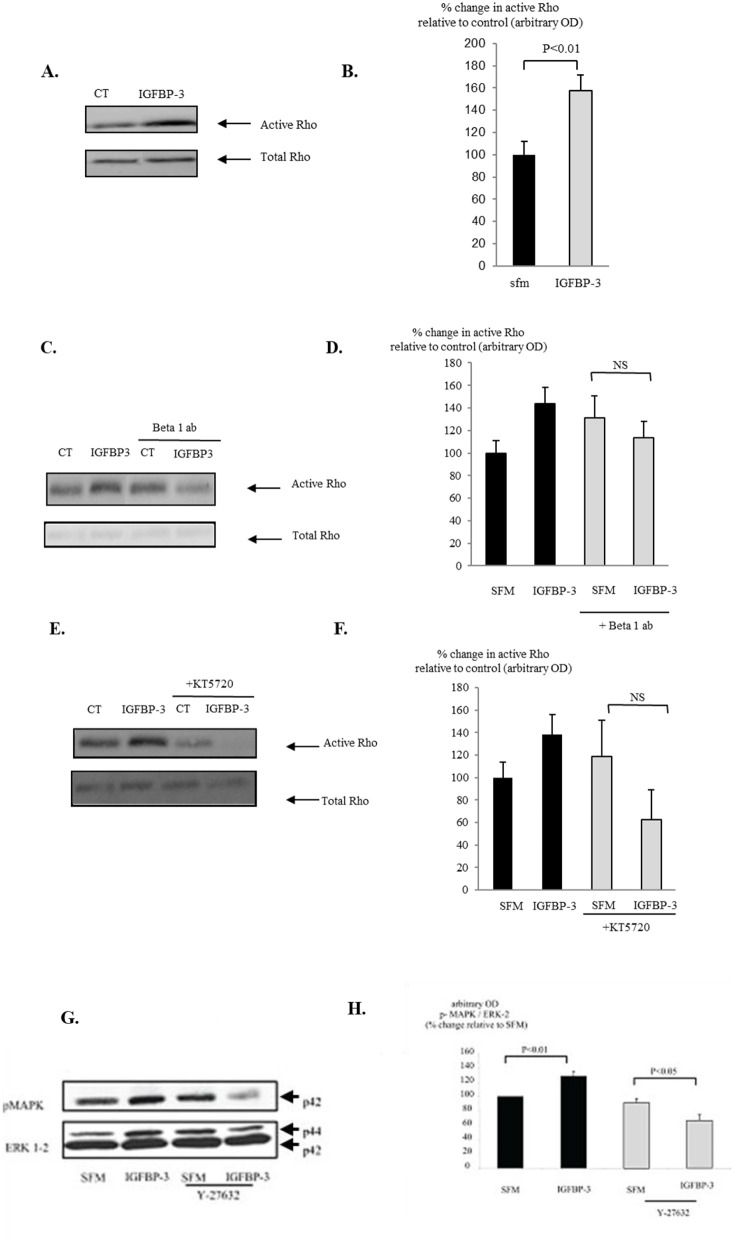
Hs578T were seeded at 0.3×10^6^ per T25 culture flasks for 24 h after which the GM was replaced with SFM for 24 h. Cells were treated **(A)** with IGFBP-3 (100 ng/ml) for 30 min or **(C)** with IGFBP-3 (100 ng/ml) following a 30-min pre-dose with an anti-beta 1 integrin blocking antibody (200 ng/ml) or **(E)** a PKA inhibitor KT5720 (1μM). Following treatment activation of Rho was assessed as described in Section “Materials and Methods.” **(A, C, E)** show Western immunoblots (of active and total Rho and are representative of experiments repeated three times) and **(B, D, F)** graphs of the mean arbitrary optical density measurements from three experiments demonstrating the change in active Rho compared to the control. Cells were treated with IGFBP-3 (100 ng/ml) for 30 min following a 30-min pre-dose with Y-27632 (5μM) and **(G)** shows Western immunoblots of p-MAPK and total ERK-2 and are representative of experiments repeated three times. The graph **(H)** shows the mean arbitrary optical density measurements from three experiments demonstrating changes in p-MAPK corrected for ERK-2.

The original version of this article has been updated.

